# The neural basis of deception in strategic interactions

**DOI:** 10.3389/fnbeh.2015.00027

**Published:** 2015-02-12

**Authors:** Kirsten G. Volz, Kai Vogeley, Marc Tittgemeyer, D. Yves von Cramon, Matthias Sutter

**Affiliations:** ^1^Werner Reichardt Centre for Integrative NeuroscienceTübingen, Germany; ^2^Department of Psychiatry and Psychotherapy University of CologneGermany; ^3^Institute for Neuroscience and Medicine – Cognitive Neuroscience (INM3), Research Center JülichJülich, Germany; ^4^Max Planck Institute for Metabolism ResearchCologne, Germany; ^5^Max Planck Institute for Human Cognitive and Brain SciencesLeipzig, Germany; ^6^Department of Public Economics, University of InnsbruckInnsbruck, Austria; ^7^Department of Economics, University of CologneCologne, Germany

**Keywords:** deception, sophisticated deception, fMRI experiment, temporo-parietal junction, strategic interactions, habenula

## Abstract

Communication based on informational asymmetries abounds in politics, business, and almost any other form of social interaction. Informational asymmetries may create incentives for the better-informed party to exploit her advantage by misrepresenting information. Using a game-theoretic setting, we investigate the neural basis of deception in human interaction. Unlike in most previous fMRI research on deception, the participants decide themselves whether to lie or not. We find activation within the right temporo-parietal junction (rTPJ), the dorsal anterior cingulate cortex (ACC), the (pre)cuneus (CUN), and the anterior frontal gyrus (aFG) when contrasting lying with truth telling. Notably, our design also allows for an investigation of the neural foundations of sophisticated deception through telling the truth—when the sender does not expect the receiver to believe her (true) message. Sophisticated deception triggers activation within the same network as plain lies, i.e., we find activity within the rTPJ, the CUN, and aFG. We take this result to show that brain activation can reveal the sender's veridical intention to deceive others, irrespective of whether in fact the sender utters the factual truth or not.

## Introduction

Communication based on informational asymmetries abounds in politics, business, and almost any other form of social interaction. Such situations may provide an incentive for either party to exploit the informational asymmetries to their own advantage. This may then imply the use of deception. Although there is some debate about a coherent and generally accepted definition, typically experimental (neuroscientific) investigations are based on a conceptual definition of deception as a deliberate act that is “intended to foster in another person a belief or understanding which the deceiver considers false […]. Specifically, the deceiver transmits a false message (while hiding the true information) […]” (Zuckerman et al., 1981, p. 3). Consider, for example, customers in a restaurant who ask the waiter if the lobster is fresh. The waiter may care only about the customers' well-being, and answer truthfully. Alternatively, she may be motivated by the restaurant's need to get rid of the less fresh lobsters and answer untruthfully. Informational asymmetries often provide an incentive for the better-informed party to exploit her informational advantage by holding back information from another party, thus involving some sort of lying or misrepresentation of information.

Yet, wrongly informing the interaction partner about the true nature of a situation is only one form of deception and excludes other important deceptive acts, such as sophisticated deception (Sutter, 2009). By taking into account the sender's thoughts about the receiver's belief, sending a true message can also be classified as a form of deception. Particularly, the sender may tell the receiver about the true state of the world, hoping she will think the sender is lying and will therefore not act according to the information provided. For instance, think of opposing parties in war. Here, a sophisticated lie would be to tell the enemy exactly what you are going to do, hoping the opponent will think you are lying and will therefore not act according to the information you provide. In contrast, a plain lie would mean sending the wrong information, such as pretending to invade the other's territory at a different location from where the attack is actually carried out. Accordingly, sophisticated deception and simple deception can be delineated along the dimensions “truth of the proposition” (true vs. false) and “the sender's belief about the receiver's expectation” (to be deceived vs. not to be deceived), whereas the intention of the sender is in both cases to deceive the receiver. In contrast, sophisticated deception can be delineated from plainly telling the truth along the dimensions “intention of the sender” (to deceive vs. not to deceive) and “the sender's belief about the receiver's expectation” (to be deceived vs. not to be deceived). Together, sophisticated deception can be thought of as some sort of a hybrid, it conveys literally the truth, but is intended (and expected) to be perceived as a lie.

In this paper, we analyze the neural foundations of simple as well as sophisticated deception in strategic interactions. Particularly, we ask whether brain activation patterns can reveal the sender's true intention and can disentangle the two forms of deception, namely simple and sophisticated deception. By using functional Magnetic Resonance Imaging (fMRI), we can derive qualitative and quantitative predictions for brain activation patterns that can help to contrast different candidate strategies that may not be evident from behavioral data alone.

As outlined above and put forward repeatedly for deception in interactive contexts (cp. meta-analysis by Lisofsky et al., 2014), the intention to deceive requires the sender to anticipate the receiver's mental state and thus think about her beliefs and expectations. Building on the notion that telling the truth is some sort of baseline (Cui et al., 2014), we propose that the intention to deceive the interaction partner, regardless of how it is expressed eventually, requires additional socio-cognitive processes than does telling the truth. This should also be reflected by longer reaction times for both sorts of deceptive behavior when compared to truth telling as well as be reflected on the phenomenological level (i.e., senders' reports). Therefore, we expect increased neural activation when comparing simple and sophisticated deception to plainly telling the truth specifically within regions that have been associated with theory of mind (ToM) processes, such as the right temporo-parietal junction (rTJP), including the posterior superior temporal gyrus/angular gyrus (Frith and Frith, 1999; Vogeley et al., 2001; Amodio and Frith, 2006; Decety and Lamm, 2007; Wolf et al., 2010) and with social cognition, such as the temporal pole (TP) (Moriguchi et al., 2006; Frith, 2007; for a review see Olson et al., 2007). The hypothesized activation pattern reflecting the intention to deceive (TPJ, TP) shall also be observed for sophisticated deception when compared to plain truth trials. Therefore, we could distinguish the two forms of sending objectively true messages and unfold the sender's true (deceptive) intention. Finding activation within areas reflecting socio-cognitive processes specifically for deceptive behavior (irrespective of how it unfolds) as compared to truth telling would be novel and taken to indicate the specific requirement of such processes for deception in strategic interactions. In other words, if the outcome of the interaction depends on both, the sender and the receiver, deceptive behavior—undertaken to get a (monetary) advantage—requires other processes than solely saying the truth and therewith accepting the outcome of the interaction without any attempts to influence it.

For plain lies (as compared to plainly telling the truth) we expect (in addition to TPJ and TP) activation within the anterior cingulate cortex (ACC). A recent quantitative meta-analysis on deceptive behavior in social interactive paradigms (Lisofsky et al., 2014) suggested this activation “to indicate greater conflict processing during deception in social situations in which people are especially supposed to behave honestly” (p. 119). This ACC activation for plain lies is expected to vary depending on the intensity of conflict, which we define as the product of the differences of the sender's and the receiver's monetary payoffs.

Taking into account the sender's true intention, allows us (for the first time) to specifically investigate the neural correlates of genuine truth trials. In none of the previous imaging studies on deception did the authors report any specific activation pattern for telling the truth. If this was due to truth trials being a heterogeneous category (for instance, including truth trials with the intention to deceive), we shall find a specific activation pattern for telling the truth in this study.

Studying deception in strategic interaction requires participants be given a choice of whether to deceive another person, because only when they have a choice can we find out the circumstances under which subjects will resort to deception (Abe et al., 2007; Greene and Paxton, 2009; Sip et al., 2010; for a review see Sip et al., 2008). For this reason, paying participants according to their choices—as is standard in experimental economics (Smith, 1976)—is important. Accordingly, in the present study participants played a simple sender-receiver game (Crawford and Sobel, 1982; Gneezy, 2005). In this two-person game, the sender (e.g., the waiter in the introductory example) is informed about two possible states of the world (the lobster is fresh or not) that yield particular payoffs for the sender and the receiver (the customer). The sender can send a message to the receiver that is either true or false with respect to which state of the world is more profitable for the receiver. Based on this message, the receiver makes a decision (whether to order the lobster or not), thus determining the payoffs for the sender and the receiver. That is, the monetary payoff for the sender highly depends on whether she is successful in making the receiver believe her. We assume the receiver cannot figure out whether the message is true (e.g., the customer cannot retaliate if he finds out the lobster was bad). This is different from a recent paper on the neural circuitry of a broken promise in which the person sending a promise was also the person making the decision about whether to keep the promise (Baumgartner et al., 2009). In our context, sending a message is the only action the sender can take and thus the only way in which she might influence the receiver. Taken together, our paradigm addresses widespread concerns around ecological validity of experiments on deception in that it is truly interactive, participants have a real opportunity to deceive another person who is not a confederate, and participants' payoffs (in the role of the sender) depend fully on the decision of the receiver. Moreover, due to the specificity that the receiver cannot find out whether the sender had sent a wrong message or not allows us to investigate deceptive behavior in strategic interactions that is unaffected by learning and adaptation effects. It is for the latter reason that we give no feedback to receivers about the actual options from which the sender could choose from.

## Materials and methods

### Participants

Thirty-four (17 women, mean age = 24.3 years, *SD* = 2.6, range = 21–32 years) right-handed^1^, healthy volunteers (without any neurological or psychiatric history) participated in the fMRI experiment for a payment of 12 Euro per hour. Additionally to this show-up fee, participants could earn up to 30 Euros. That is, at the end of the experiment, one trial was randomly drawn and paid out according to the receiver's choice in this specific trial. All participants had normal or corrected-to-normal vision, spoke German as their native language, and none had irremovable metal implants in their bodies. The experimental procedure and data collection followed the ethical guidelines of the “Declaration of Helsinki” (revised version, 2012) and were approved by the local ethical committee of the University of Cologne. Data were handled anonymously. We had to exclude four participants (1 male and 3 female) from the analysis because of too few lying or sophisticated deception trials, respectively, and one participant because of zero truth trials.

### Stimuli and experimental paradigm

**Figure 1:**
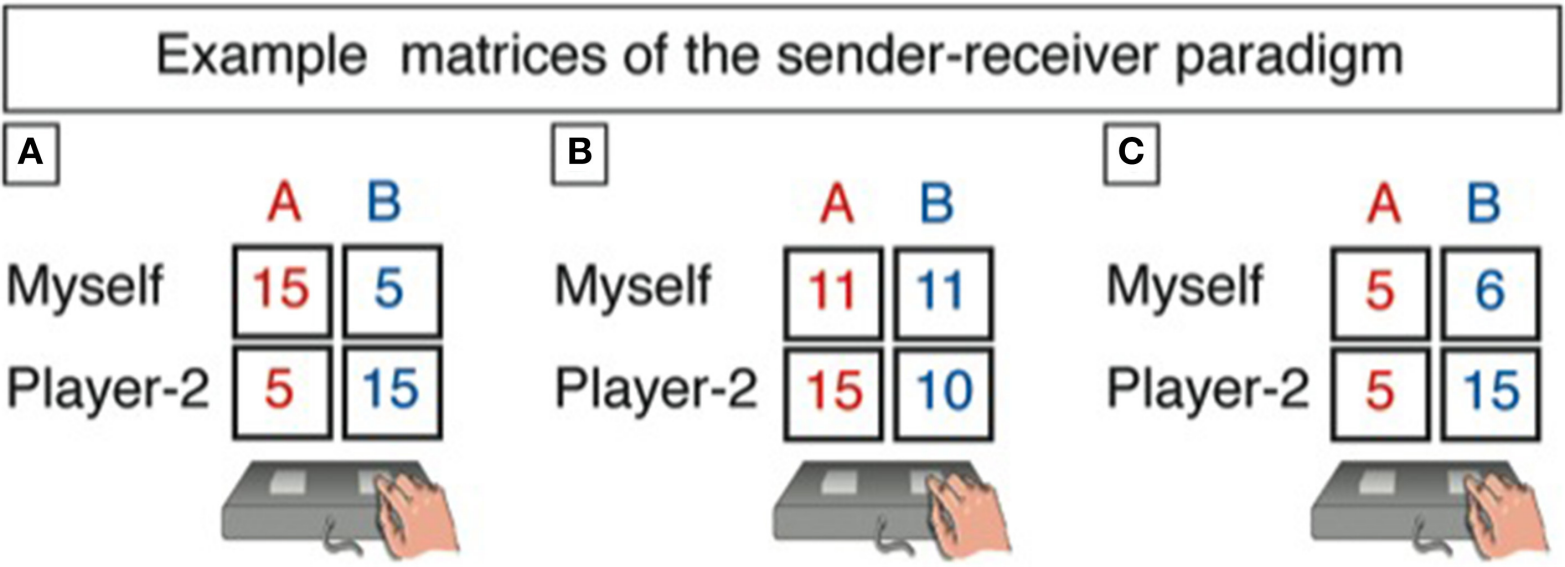
**This is how we presented the payoffs in the two states of the world to the sender**. Tables A1–A3 in the Appendix list all 90 games. Example matrices of the sender-receiver paradigm are given for the three conditions “conflict” **(A)**, “sender indifferent” **(B)**, and “aligned interest” **(C)**. The sender is shown a specific payoff matrix and can send either of two messages: “Red is more profitable for you.” Or “Blue is more profitable for you.” After response selection and on the next screen, the participant has to answer the following question: “Which state do you expect the receiver to choose? The red column or the blue column?” Importantly, the sender's message does not have a direct impact on the payoffs for both players in any of the states. Rather, the receiver's choice is eventually implemented for payment.

In the sender-receiver game, there are two players of which only the sender (the person being scanned) is informed about the monetary consequences for herself and the receiver for two different options, one being associated with Blue color and the other with Red color. Let Blue (S^b^, R^b^) represent the payoff to the sender and the receiver, respectively, from choosing Blue, and Red (S^r^, R^r^) from choosing Red (cp. Figure 1). After being informed about these pairs of payoffs, the sender sends a message to the receiver, saying either “Blue is more profitable for you” or “Red is more profitable for you.” After sending a message, the sender has to indicate on a new screen which state she expects the receiver to pick. Then the next trial started. All in all, 90 games were played that differed with respect to the relative gains and losses for the two players (see below).

We call a choice a ***sophisticated deception*** when a sender sent the true message expecting the receiver *not* to follow it. We denote as a ***true message*** a case in which a sender sent the true message and expects the receiver to follow it by picking the state the message indicated as more profitable. We classify as ***simple deception*** cases in which the sender sent the false message. After receiving the message from the sender, the receiver chooses Blue or Red, and the respective payoffs are recorded (cp. Table 1).

**Table 1:** **Performance refers to the answer to the first question: “Which option (blue or red) is more profitable for Player 2?”; intention to deceive refers to the answer to the second question: “Which state do you expect the receiver to choose? The red column or the blue column?**”

**Performance: honest answer?**	**Intention: intention to deceive?**	**Trial classification**
Yes	No	Plain truth
	Yes	Sophisticated deception
No	Yes	Simple deception
	No	Not classifiable (ignored)

While the sender underwent the anatomical scanning session (to obtain the individual anatomical structures onto which the metabolic activity map was projected), the receiver played the game in another room, which was located across campus, and it was ensured that sender and receiver never met each other. This was done to exclude any effects that might arise as a consequence of attractiveness, sympathy, gender, or the like. After the receiver finished her part (which was approximately at the time the scanning session of the sender was finished), one trial was randomly picked by the experimenter and the corresponding payoff (additional to the show-up fee of 12 Euros/h) was paid out to the sender and the receiver according to the receiver's choice. The mean additional payout for senders was €8.53 (*SD* = 5.54), ranging from 5 to 20 Euros; the mean additional payout for receivers was €8.26 (*SD* = 6.15), ranging from 0 to 25, not being significantly different [*t*_(58)_ = 0.176, *p* = 0.861]. The full set of instructions is provided in the Appendix and both, sender and receiver, knew about the entire procedure before starting the experimental session (see A.1 and A.2 in Supplementary Material).

In each of the 90 games, the sender was asked to send one of the above messages to the receiver. One of these messages was always true and the other was false. Knowing only the message she received and not the potential payoffs in each state, the receiver had to pick either Blue or Red, which then determined the payoffs for the sender and the receiver. Since the receiver was only informed about her actual payoff in the chosen state—and not about the sender's actual payoff or the possible payoffs in the un-chosen state—the receiver could not judge whether the sender had told the truth or not. Yet, the receiver was informed that the maximum profits for her and the sender was 30 Euros. It was important that the receiver did not know about the potential payoffs in each state (but only the payoff of the actually chosen option in the current trial), otherwise she would have adjusted her behavior, thus confounding objectivity and comparability (within and across participants) as well as affecting the sender's strategic behavior. Likewise, to exclude learning and order effects on the side of the sender's behavior, the sender did not learn about the decisions of the receiver.

We varied the incentives for deception along three different categories for the 90 games, indicating the possible tension between the sender's and receiver's payoffs (i.e., stimulus-dependent categorization independent of participants' choice). In the category “***conflict***” (*n* = 45), the more profitable state for the sender was always less profitable for the receiver. We also varied the relative gains and losses of the sender and the receiver between the two states of a game. In category “***sender indifferent***” (*n* = 27), the sender earned the same amount of money in both states, but the receiver payoff differed across states, and it could be higher or lower than the sender's payoff. Category “***aligned interests***” (*n* = 18) included only pairs of states in which one state yielded higher profits both for the sender and the receiver, although the increase in payoffs from the worse to the better state could differ for sender and receiver. The order of presentation of games was randomized. The full set of games is provided in the Appendix (see A.3 in Supplementary Material).

All trials lasted for 16 s (i.e., 8 scans at TR = 2 s): the game with its monetary payoffs was presented for a maximum of 8 s, during which time participants could respond, followed by a short fixation (2 s) and then the question about the sender's expectation (4 s). Subsequently, the announcement that the next trial was about to start was presented for 2 s. To help to characterize the shape of the hemodynamic response function, the timing of the presentation of the stimulus was varied. Accordingly, using a jittering-method more points of the hemodynamic response function can be sampled than if a fixed inter-stimulus-interval was used. Particularly, we randomly varied the onset of each stimulus presentation relative to the beginning of the first of the eight scans (0, 400, 800, 1200, 1600 ms) to enhance the temporal resolution of the signal captured (Miezin et al., 2000; Birn et al., 2002).

### MR scanning procedure

#### Image acquisition

Imaging was performed on a 3T scanner (Siemens TRIO, Erlangen, Germany) equipped with a standard birdcage head coil. Participants lay supine in the scanner with their hands placed on a right and left response button box. The index fingers were placed on two appropriate response buttons and participants were trained about the response contingencies. Form-fitting cushions were used to prevent participants from head movement and they were provided with earplugs to attenuate the scanner noise. The experiment was presented via a mirror that was mounted to the headcoil and individually adjusted.

One of the areas, in which we expected activation, is the TP. This area is subject to severe distortion and signal loss in fMRI due to susceptibility artifacts that result from the area's specific location, i.e., near air-filled sinuses (Ojemann et al., 1997). Therefore, we used a spin-echo (SE) sequence which has been shown to be less sensitive to susceptibility-related signal dropouts as in contrast to gradient-echo (GE) sequences (Norris et al., 2002; Schmidt et al., 2005). Yet, the drawback of using SE-based instead of GE-based fMRI is a lower statistical power of the SE sequences.

During functional imaging, 17 axial slices (4 mm thickness, 25% spacing, field of view [FOV] 21 cm, data matrix of 64 × 64 voxels, and in-plane resolution of 3.3 × 3.3 mm) covering the whole brain were collected using a single-shot SE echo-planar imaging (SE-EPI) sequence (TR 2 s, echo time [TE] 80 ms, flip angle 90°) sensitive to blood oxygen level-dependent (BOLD) contrast. One functional run with 728 timepoints was run with each time point sampling over the 17 slices. After the functional imaging, high-resolution 3D T1-weighted whole brain MDEFT sequences (128 sagittal slices, 1 mm thickness) were recorded.

#### Image processing and analysis

The functional imaging data were processed and analyzed using the software package LIPSIA (Leipzig Image Processing and Statistical Inference Algorithms) version 2.2 (Lohmann et al., 2001). To correct for temporal offsets between the slices acquired in one scan, a cubic-spline interpolation was used. Thereafter the data were motion-corrected with the 50th time-step as a reference and 6 degrees of freedom (3 translational, 3 rotational). A temporal high-pass filter with a cutoff frequency of 1/120 Hz was used to remove low-frequency signal changes and baseline drifts and a spatial Gaussian filter with 6 mm full-width half-maximum (FWHM) was applied. A rigid linear registration with 6 degrees of freedom (three rotational, three translational) was performed to align the functional data slices with a 3D stereotactic coordinate reference system. The rotational and translational parameters were acquired on the basis of the MDEFT slices to achieve an optimal match between these slices and the 3D reference data dataset. The MDEFT volume data was standardized to the MNI atlas. The rotational and translational parameters were subsequently transformed by linear scaling to the same standard size. The resulting parameters were then used to transform the functional slices employing a trilinear interpolation, so that the resulting functional slices were aligned with the stereotactic coordinate system. Resulting data had a spatial resolution of 3 × 3 × 3 mm (27 mm^3^).

The statistical evaluation was based on a least-squares estimation using the general linear model (GLM) for serially auto-correlated observations (Friston et al., 1995; Worsley and Friston, 1995). The design matrix was generated with a delta function, convolved with the hemodynamic response function (gamma function) (Glover, 1999). We used two different design matrices to answer the different research questions. One design matrix comprised the following events: truth trials, simple deception trials, and sophisticated deception trials (cp. Table 1). The trials were classified based on participants' behavior, i.e., their choice which message to send to the receiver and their response to the question “Which state do you expect the receiver to choose?” Events were modeled time-locked to the beginning of a game. The duration was modeled individually with the time it took participants to respond to the game (RT) (Grinband et al., 2008) and with amplitude of one. In another design matrix that was used to model and investigate the effects of conflict (defined as the tension between the sender's and receiver's payoffs), we had five regressors, particularly, truth trials, simple deception trials, and sophisticated deception trials with their duration being modeled individually by RT and amplitude of one plus two regressors for simple deception trials and sophisticated deception trials that were modeled with their individual RT and an amplitude that reflected the tension between the sender's and receiver's payoffs. The tension to deceive was calculated as the product of the differences of the sender's and the receiver's payoff for the pairs of states, i.e., (S^b^ – S^r^)^*^(R^r^ – R^b^) (cp. description of stimulus material and Figure 1). For instance, let S^b^ = 15, S^r^ = 5, R^r^ = 15, and R^b^ = 5, then the value representing the tension between the player's payoffs is (15 – 5) × (15 – 5) = 100. In contrast, for a matrix with the payoffs S^b^ = 1, S^r^ = 0, R^r^ = 5, and R^b^ = 0, the conflict value is low ((1 – 0) × (5 – 0) = 5). This value represents the product of the difference of the profit of the sender and the corresponding inverted difference of the receiver. This means that if the differences have opposite signs, then the sender and the receiver have conflicting interests. In case the differences have the same sign, both the sender and the receiver gain higher profits in the same state. If the sender is indifferent between the two states, the parameter value is zero. Hence, this conflict parameter reflects a measure of the tension to deceive.

For each participant, contrast images were generated on the basis of beta-value estimates of the raw-score differences between specified conditions. Subsequently, these single subject contrasts were entered into a second-level analysis on the basis of Bayesian statistics (Neumann and Lohmann, 2003; Lazar, 2008). In the approach by Neumann and Lohmann (2003), posterior probability maps and maps of the effect size are calculated on the basis of the resulting least-square estimates of parameters for the GLM. That is, the parameter estimates on the second level of analysis are viewed within a Bayesian framework as evidence for the presence or absence of the effect of interest in a group of participants. The output of the Bayesian second-level analysis is a probability map showing the probability for the contrast to be larger than zero. This Bayesian technique allows us to directly estimate the probability of a specific difference in the group means given the parameter estimates of the GLM for the individual participants. This is more informative than a classical rejection of a null hypothesis. This approach has the further advantage, when compared with conventional analyses based on *t* statistics, of being less sensitive to outliers than traditional *t* statistics, as the influence of individual participants on a group statistic is weighted by the within-subject variability. In support of this, Thirion et al. (2007) suggested that, from the point of view of reliability, optimal statistical thresholds for activation maps are lower than classical thresholds corrected for multiple comparisons. Furthermore, since probabilities of the contrasts are calculated, but no significance tests are performed, corrections for multiple comparisons or calculations of effect sizes are not necessary. For visualization, a threshold of 99.4% was applied to the probability maps.

## Results

### Behavioral results

As expected, the frequency of sending the false message strongly depends on a game's category, i.e., on the distribution of payoffs (for a description of the stimulus-dependent categorization please see Stimuli and Experimental Paradigm): it is fairly low in the “*aligned interest*” category (25%, *SD* = 22.5) and in “*sender indifferent*” (24.7%, *SD* = 23.2), but comparatively high in “*conflict*” (60.8%, *SD* = 21.5) [*F*_(2, 28)_ = 34.97, *p* = 0.0001]. Lying in the “*conflict*” category is significantly more frequent than in either “*aligned interest*” or “*sender indifferent*,” whereas we find no significant difference between the latter two categories. Furthermore, the possible gains for the sender if the receiver picks the state that is better for the sender, and the potential losses for the receiver if she picks the state that is worse for her, have a significant impact on the likelihood of sending the false message. Senders lie more often when the potential gains from lying are high (10€ or 5€; 55.8%, *SD* = 19.2) than when they are low [1€ or 0€; 34.2%, *SD* = 17.2; *t*_(29)_ = 6.1, *p* = 0.0001]. Senders lie less often when the possible losses for the receiver are high (10€, 15€, or 20€; 37.4%, *SD* = 20.3) than when they are low [1€ or 5€; 47.2%, *SD* = 13.3; *t*_(29)_ = −4.04, *p* = 0.0001]. These results clearly indicate that monetary incentives affect the frequency of sending the false message.

The relative frequency of sophisticated deception (as a fraction of the total number of cases in which the sender sent the objectively true message) depends on a game's category in the same way the frequency of simple deception does. In the category “*conflict*,” we observe sophisticated deception in 59.3% (*SD* = 31.5) of cases with true messages, whereas we observe it significantly less often in “*sender indifferent*” (40.9%, *SD* = 28.6) and “*aligned interest*” [31.7%, *SD* = 19.1; *F*_(2, 26)_ = 14.98, *p* = 0.0001]. This finding indicates sophisticated deception through telling the truth is most likely when the sender can profit most from it. Adding the cases of sophisticated deception to the cases of simple deception, the overall frequency of deception reaches 65.9% (*SD* = 18.9) across all categories, whereas it is only 42.8% (*SD* = 15.3) when taking into account only plain lies and ignoring deceptive behavior through truth telling.

Our assumption that truth telling may be less demanding than deceiving the interaction partner was confirmed for both sorts of lying: Telling a plain lie (*M* = 2618 ms, *SD* = 202) or engaging in sophisticated deception (*M* = 2611 ms, *SD* = 193)—while not significantly different from each other—takes significantly longer than telling the truth (*M* = 2453 ms, *SD* = 211) [*F*_(2, 30)_ = 3.46, *p* = 0.044]. This response pattern is crucially affected by the actual payoffs: A 3 (category) × 3 (response (truth, SD, plain lies)) repeated measures ANOVA reveals a significant main effect of category [*F*_(2, 30)_ = 6.44, *p* = 0.005], with “conflict” trials showing the longest RTs (*M* = 2720 ms, *SD* = 211) followed by “sender indifferent” trials (*M* = 2565 ms, *SD* = 203) and “aligned interest” trials (*M* = 2397 ms, *SD* = 200) (cp. Table 2). We take these results to support the notion that deceptive behavior, irrespective of how it is expressed, demands additional cognitive processes so as to suppress a pre-potent truthful answer. This is also supported by our post-session questionnaire data: senders report that it took them significantly longer to respond when stakes were high and that they had to deliberate harder when preparing to deceive the receiver.

**Table 2:**
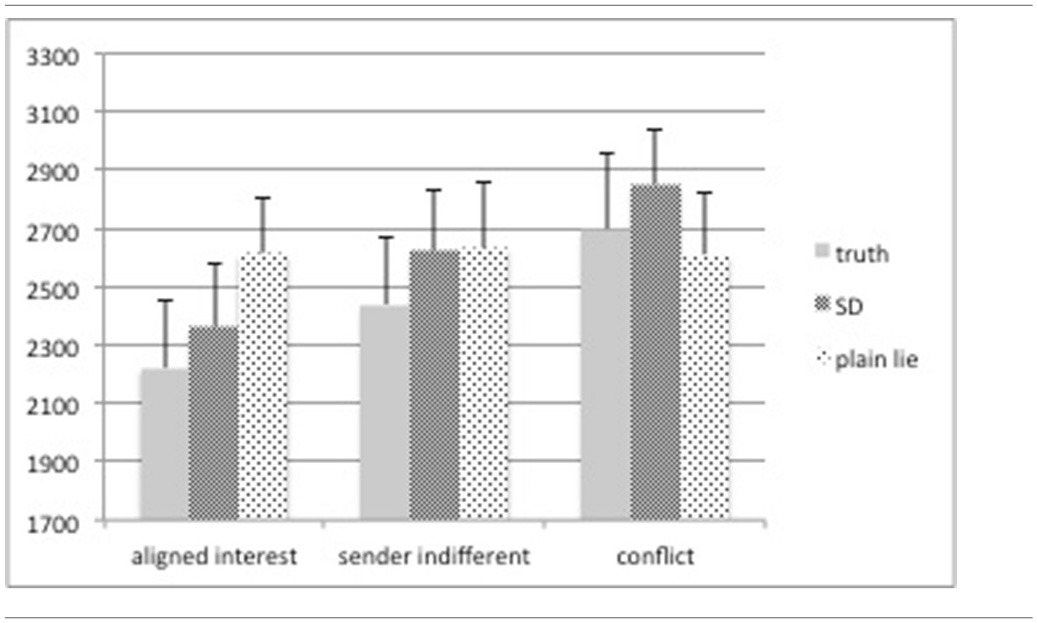
**Reaction times (in ms) split by category (“aligned interest,” “sender indifferent,” and “conflict,” please cp. section on stimuli and experimental paradigm for more details) and deceptive behavior [truth, sophisticated deception (SD), and plain lies]**.

Additional results from the post-session questionnaire data reveal insights regarding strategy and heterogeneity. Concerning the former, 86.6% of the senders report having developed a strategy how to interact with the receiver and of those more than half (59.9%) report that their strategy depended on the difference in payoffs between sender and receiver as well as on the absolute amounts. The remaining senders indicate to have taken into account the frequency and succession of previous blue- and red-responses so as to determine how to respond. We take these findings to indicate that senders engaged, indeed, in our social interactive paradigm and cared about the actual payoffs. Concerning the issue of heterogeneity, the data display a heterogeneous sample. Being asked on how many of the trials they produced a deceptive response, senders on average say that they did so in 43.8% (*SD* = 23) of the cases, the range being 5–90%. A closer look reveals that 36.6% of the senders have had a bad conscience when producing a deceptive response (with the feeling even persisting for a couple of trials) and feel that they had lied in effect. These senders indicate to have lied in only a third of the trials (*M* = 33.4%, *SD* = 21.6). In contrast, the other senders (63.3%) report not having had a feeling of actually lying, and thus indicate having lied in approximately half of the trials [*M*= 49.7%, *SD* = 22.1, *t*_(28)_ = −1.95, *p* = 0.06 (2-tailed)].

### Imaging results

#### Neural correlates of the intention to deceive in strategic interactions (simple and sophisticated deception > truth)

**Figure 2:**
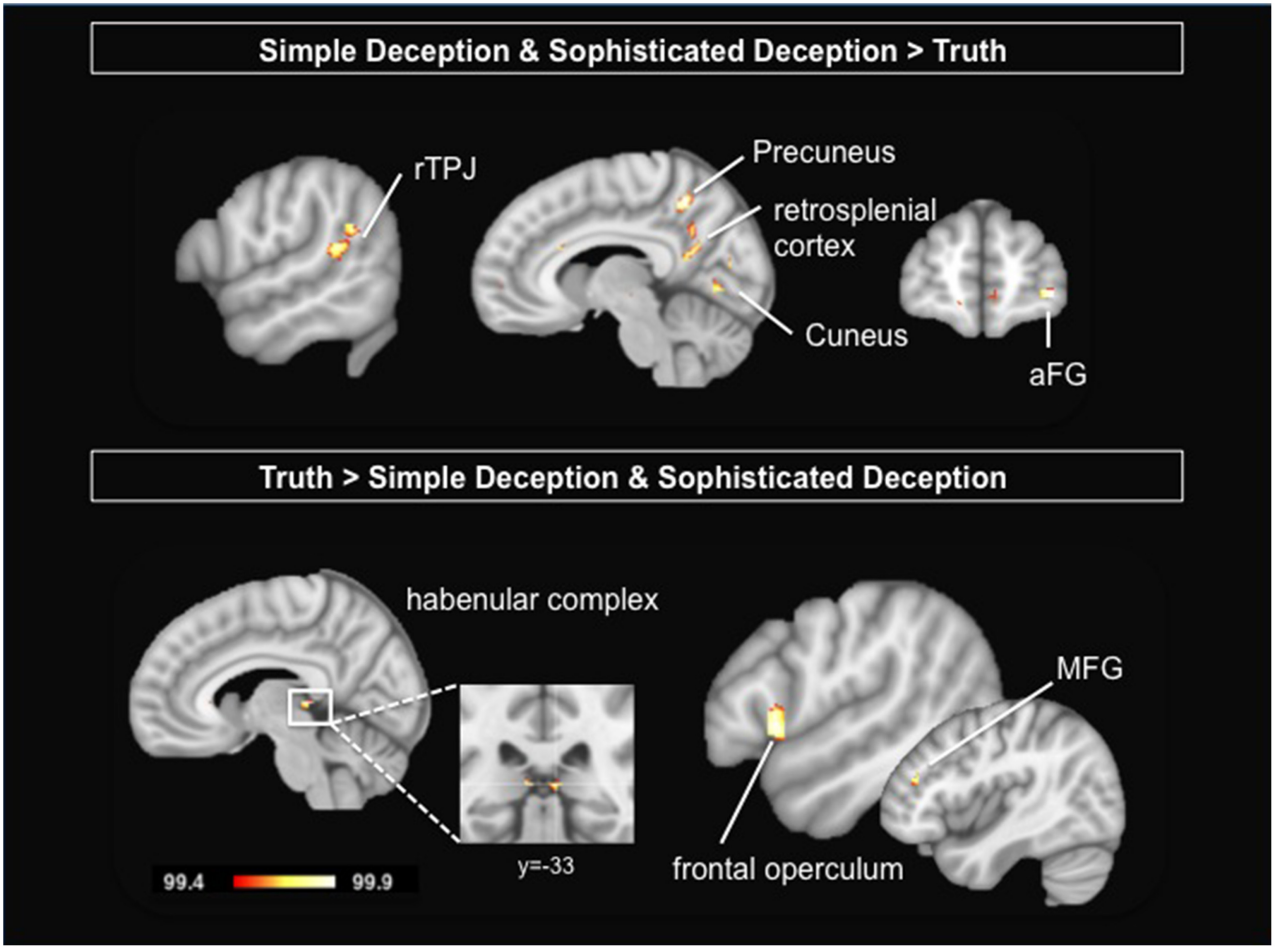
**Upper Panel:** Intention to deceive in strategic interactions: Results are shown for the contrast simple deception *and* sophisticated deception trials vs. truth trials. **Lower Panel:** Telling the truth: Results are shown for the contrast truth trials vs. simple deception *and* sophisticated deception trials. Abbreviations: aFG, anterior frontal gyrus; MFG, middle frontal gyrus; rTPJ, right temporo-parietal junction. For visualization, a threshold of 99.4% was applied to the probability maps.

To study the neural correlates of the intention to deceive, we contrast the hemodynamic activation of simple deception trials *and* sophisticated deception trials with truth trials and find activation within the right TPJ, superior temporal gyrus, precuneus extending into the retrosplenial cortex, cuneus bilaterally, and within the right superior frontal gyrus (BA 10) (see Table 3 and Figure 2, upper panel).

**Table 3:** **Intention to deceive in strategic interactions: laterality, anatomical specification, Talairach coordinates (*x*, *y*, *z*), posterior probabilities, and size (mm^3^) for activations according to Bayesian analysis are shown for the contrast simple deception *and* sophisticated deception trials vs. truth trials**.

**Brain region**	***x***	***y***	***z***	**Max**	**mm^3^**
R. Temporo-parietal junction (TPJ)	55	−42	17	99.92	108
R. Superior temporal gyrus	43	−27	6	99.99	270
R. Precuneus	6	−51	48	99.99	648
Extending into the retrosplenial cortex	6	−57	20	99.97	189
R. Cuneus	6	−72	−2	99.97	162
	−9	−81	15	99.99	783
	−3	−69	9	99.96	189
R. Superior frontal gyrus (BA 10)	35	57	−2	99.99	216

#### Neural correlates of lying in strategic interactions (simple deception > truth)

**Figure 3:**
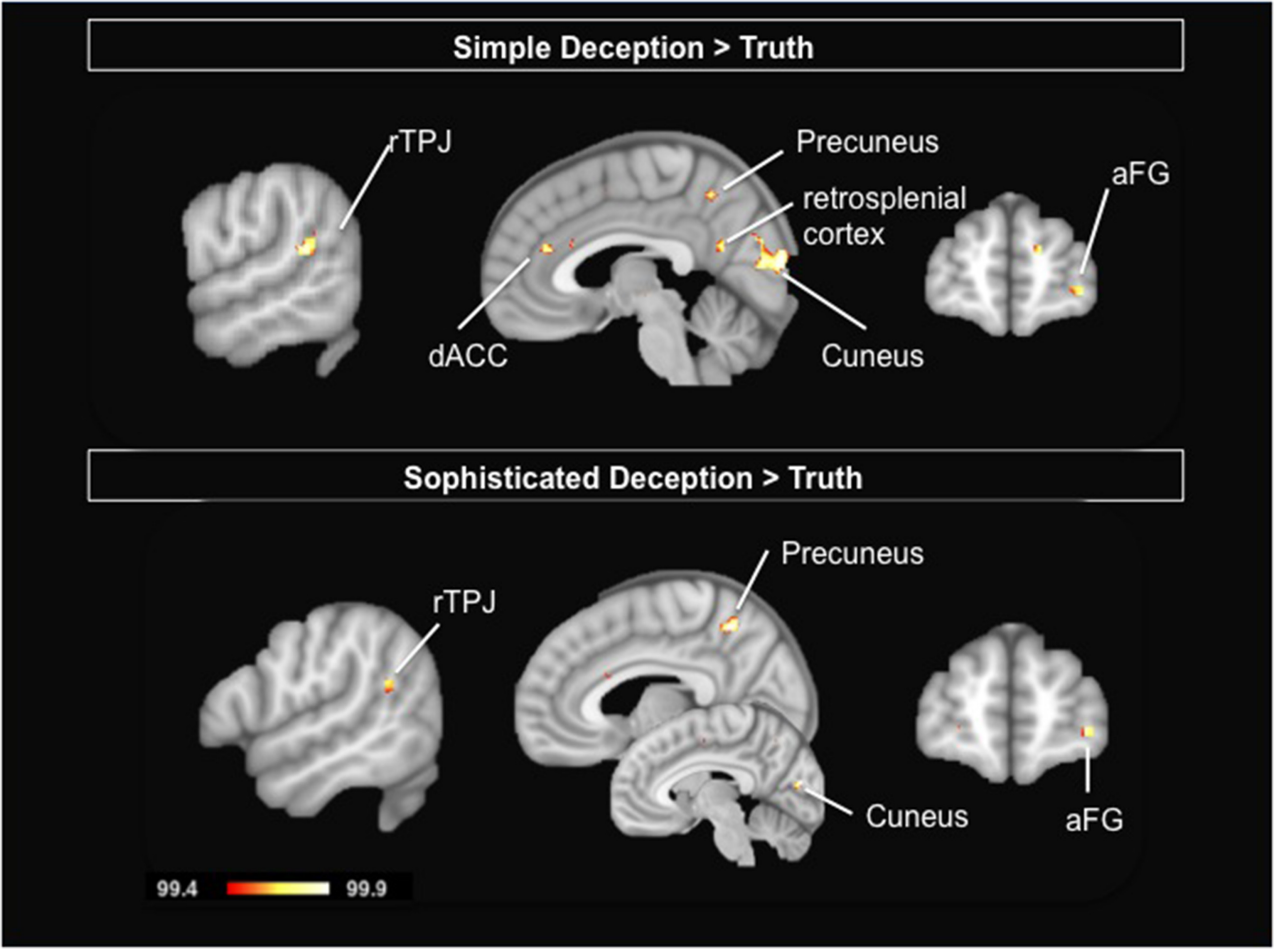
**Upper Panel:** Simple Deception: Results are shown for the contrast simple deception trials vs. truth trials. **Lower Panel**: Sophisticated Deception: Results are shown for the contrast sophisticated deception trials vs. truth trials. Abbreviations: aFG, anterior frontal gyrus; dACC, dorsal anterior cingulate cortex; rTPJ, right temporo-parietal junction. For visualization, a threshold of 99.4% was applied to the probability maps.

To study the neural correlates of simple deception, i.e., sending a false message with the intention to deceive, we contrast the hemodynamic activation of simple deception trials with truth trials and find activation within the right TPJ, the dorsal ACC, the precuneus extending into the retrosplenial cortex, within the cuneus, the right anterior frontal gyrus (aFG), and a comparatively small activation focus within the anterior medial prefrontal cortex (amPFC) (see Table 4 and Figure 3, upper panel).

**Table 4:** **Simple deception vs. truth: laterality, anatomical specification, Talairach coordinates (*x*, *y*, *z*), posterior probabilities, and size (mm^3^) for activations according to Bayesian analysis are shown for the contrast simple deception trials vs. truth trials**.

**Brain region**	***x***	***y***	***z***	**Max**	**mm^3^**
R. Temporo-parietal junction (TPJ)	58	−42	17	99.98	648
R. Anterior cingulate cortex (ACC)	3	36	23	99.87	162
R. Precuneus	6	−54	48	99.99	540
Extending into the retrosplenial cortex	6	−60	23	99.99	
R. Cuneus	6	−90	15	99.99	6021
R. Superior frontal gyrus (BA 10)	14	60	17	99.96	243
	35	57	−2	99.86	162
R. Anterior median prefrontal cortex (amPFC)	6	54	4	99.87	108

#### Neural correlates of sophisticated deception (sophisticated deception > truth)

To study the neural correlates of sophisticated deception specifically, we built a contrast of sophisticated deception trials and truth trials. We find activation within the right TPJ, the precuneus, the left cuneus, the right aFG (BA 10), and the superior temporal gyrus (see Table 5 and Figure 3, lower panel).

**Table 5:** **Sophisticated deception vs. truth: laterality, anatomical specification, Talairach coordinates (*x*, *y*, *z*), posterior probabilities, and size (mm^3^) for activations according to Bayesian analysis are shown for the contrast sophisticated deception trials vs. truth trials**.

**Brain region**	***x***	***y***	***z***	**Max**	**mm^3^**
R. Temporo-parietal junction (TPJ)	55	−51	23	99.86	162
R. Precuneus	6	−54	50	99.99	1188
L. Cuneus	−6	−81	15	99.99	1107
R. Superior frontal gyrus (BA 10)	32	57	1	99.91	216
R. Superior temporal gyrus	43	−27	6	99.86	162

Importantly, this finding suggests sophisticated deception is not a variant of plainly telling the truth—in which case no activation differences in this contrast should have occurred—but a version of telling a lie, since a very similar activation pattern occurred as in the contrast “simple deception vs. truth” (cp. upper panel in Figure 3).

#### Neural correlates delineating the two forms of deception (sophisticated deception > simple deception)

**Figure 4:**
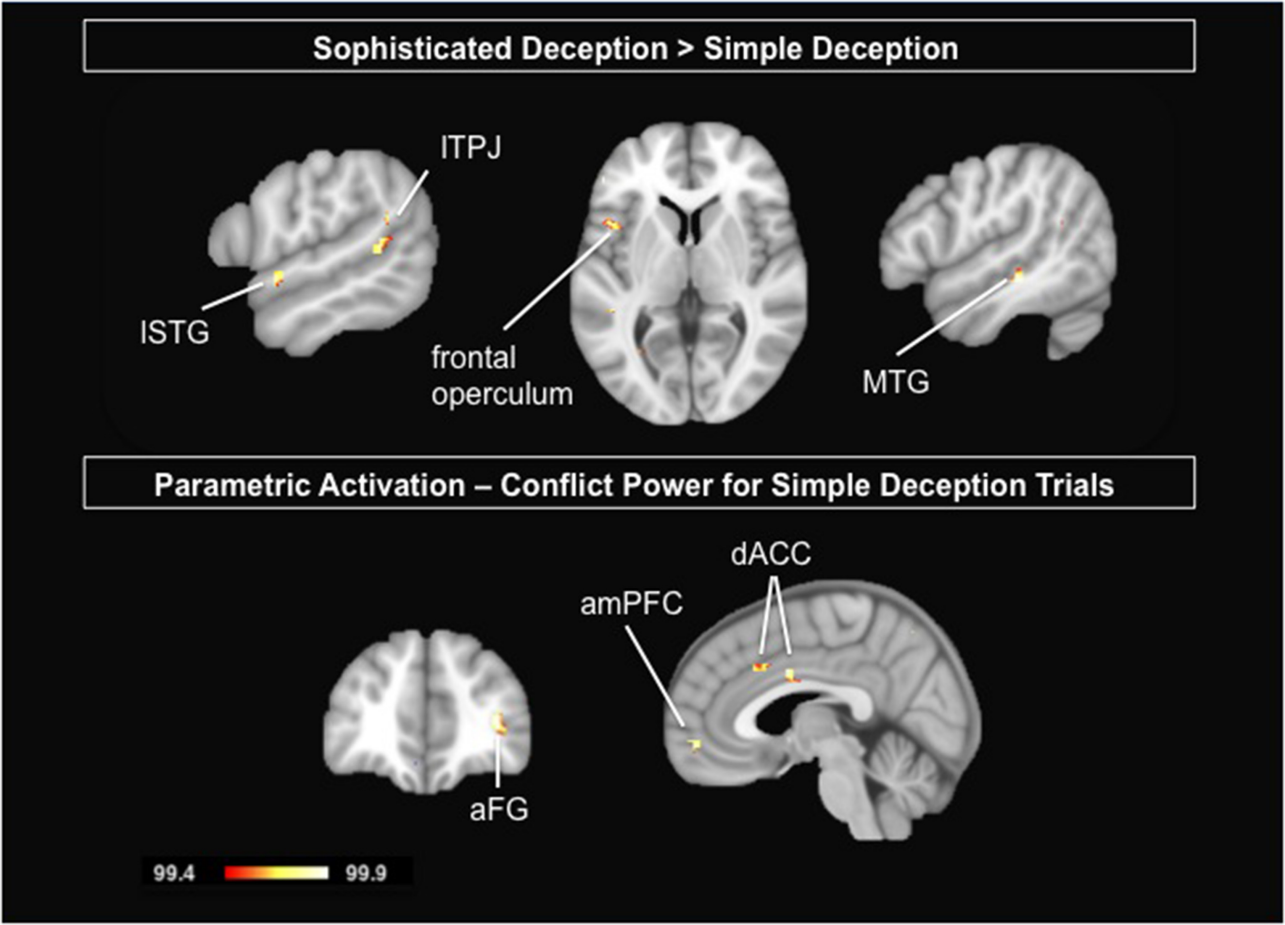
**Upper Panel:** Delineating the two forms of deception: Results are shown for the contrast sophisticated deception trials vs. simple deception trials. **Lower Panel**: Parametric analysis modeling the incentive to deceive for simple deception trials: Results are shown for the positive correlational analysis, i.e., the activation is stronger the higher the conflict and thus the tension in payoffs between sender and receiver. Abbreviations: aFG, anterior frontal gyrus; amPFC, anterior median prefrontal cortex; dACC, dorsal anterior cingulate cortex; lSTG, left superior temporal gyrus; lTPJ, left temporo-parietal junction; MTG, middle temporal gyrus. For visualization, a threshold of 99.4% was applied to the probability maps.

To test for the differences between the two forms of deception, we contrasted sophisticated deception trials with simple deception trials. We find activation bilaterally within the TPJ, the right middle temporal gyrus, the left superior temporal gyrus, the left frontal operculum, and within the mid-cingulate gyrus (see Table 6 and Figure 4, upper panel).

**Table 6:** **Sophisticated deception vs. simple deception: laterality, anatomical specification, Talairach coordinates (*x*, *y*, *z*), posterior probabilities, and size (mm^3^) for activations according to Bayesian analysis are shown for the contrast sophisticated deception trials vs. simple deception trials**.

**Brain region**	***x***	***y***	***z***	**Max**	**mm^3^**
R. Temporo-parietal junction (TPJ)	43	−60	12	99.82	189
L.	−55	−48	12	99.90	270
R. Middle temporal gyrus (MTG)	49	−27	−7	99.97	432
L. Superior temporal gyrus (STG)	−55	0	−2	99.90	243
L. Insula	−40	10	6	99.92	432
R. Mid-cingulate gyrus	6	0	42	99.98	432

#### Neural correlates of genuine truth trials

Taking into account the sender's true intention, we are able to extract genuine truth trials, i.e., trials where the sender sent the true message with the expectation that the receiver believes her (true) message. These trials are contrasted with both simple deception as well as sophisticated deception trials. We find activation within the habenular complex bilaterally, the right frontal operculum, the left pregenual ACC, and the right middle frontal gyrus (see Table 7 and Figure 2, lower panel).

**Table 7:** **Truth vs. simple and sophisticated deception: laterality, anatomical specification, Talairach coordinates (*x*, *y*, *z*), posterior probabilities, and size (mm^3^) for activations according to Bayesian analysis are shown for the contrast truth trials vs. simple deception *and* sophisticated deception trials**.

**Brain region**	***x***	***y***	***z***	**Max**	**mm^3^**
R. Habenular complex	6	−33	6	99.97	81
L.	−9	−30	6	99.86	108
R. Operculum	49	15	4	99.98	351
L. Pregenual anterior cingulate cortex	−3	33	4	99.96	135
R. Middle frontal gyrus	41	36	20	99.92	108

#### Parametric analysis modeling the incentive to deceive for simple deception trials

To test whether the activation that revealed for simple deception varies with the monetary incentive, we calculate a parametric analysis. Responses are modeled by a value that reflects the tension between the sender's and the receiver's payoffs. It is calculated as the product of the differences of the sender's and the receiver's payoff for the pairs of states, i.e., (S^b^ – S^r^) × (R^r^ – R^b^) (cp. MR Scanning Procedure). The posterior probability maps of this parametric analysis reveals the anterior median prefrontal cortex (amPFC), the dorsal ACC, and the aFG (BA 10) to be more engaged the higher the conflict and thus the tension in payoffs between sender and receiver (see Table 8 and Figure 4, lower panel).

**Table 8:** **Parametric analysis modeling the incentive to deceive for simple deception trials: laterality, anatomical specification, Talairach coordinates (*x*, *y*, *z*), posterior probabilities, and size (mm^3^) for activations according to Bayesian analysis are shown for the parametric contrast modeling the tension between the sender's and receiver's payoff in simple deception trials**.

**Brain region**	***x***	***y***	***z***	**Max**	**mm^3^**
R. Anterior median prefrontal cortex (amPFC)	3	54	−5	99.96	297
R. Anterior cingulate cortex (ACC)	6	15	37	99.96	459
R. Middle frontal gyrus (BA 10)	35	42	12	99.97	378

## Discussion

Many real life situations are characterized by informational asymmetries among interacting parties. Obviously, such situations may provide an incentive for either party to exploit the informational asymmetries to their own advantage. This may then imply the use of deception. In this fMRI study we analyze the neural foundations of deception in strategic interactions. Notably, in our paradigm, interaction partners were free whether or not to lie. Besides plain lying, we study a broader concept of deception by looking at what has been called sophisticated deception (Sutter, 2009). Here, telling the truth is counted as an act of deception when the sender expects the receiver not to follow the sender's (true) message. Moreover, by taking into account the sender's true intention, we can also determine the neural correlates of genuine truth trials. All in all, we take our results to show that brain activation patterns can reveal the sender's true intention (to deceive), for instance when sending an objectively true message.

### Intention to deceive

Particularly, our results reveal the rTPJ, the (pre)cuneus (CUN), retrosplenial cortex, and aFG to be specifically involved for the intention to deceive, irrespective of whether this is done by sending a false or a true message. The finding of activation within the rTPJ is in line with our hypothesis. Based on previous findings and recent meta-analytic findings on deceptive behavior, we suggest this activation to reflect socio-cognitive processes during deception. Specifically, deceptive behavior crucially depends on the ability to anticipate the receiver's mental state. The rTPJ, including posterior superior temporal and angular gyrus, have repeatedly been shown to be specifically involved when people have to integrate socially relevant information and to infer the mental states of others (Saxe and Kanwisher, 2003; Decety and Grèzes, 2006; Saxe, 2006; Decety and Lamm, 2007; Bahnemann et al., 2010). Thus, the finding of rTPJ activation for deceptive behavior, realized either by telling a lie or telling the truth, is consistent with our hypothesis on the intentional aspects of deception in a social setting, in which the intentional states of others are integrated into one's own reasoning (Saxe and Kanwisher, 2003; Grèze et al., 2004; Walter et al., 2004; Perner et al., 2006; Saxe, 2009).

Activation within the cuneus, precuneus, and aFG were not expected specifically but cuneus activation may reflect increased requirements as to early visual processing (Vanni et al., 2001), e.g., when thoroughly inspecting the payoff matrix, that is then sent to several parietal areas (Fattori et al., 2009); precuneus activation may reflect increased episodic memory retrieval processes (Cavanna and Trimble, 2006), for instance, retrieving past payoff matrices and one's choices in the sender-receiver game, as well as automatic social monitoring processes when observing interacting people (Iacoboni et al., 2004; Leube et al., 2012; Vrticka et al., 2013). And activation within the aFG may reflect the integration of the outcomes of two separate cognitive operations in the pursuit of a higher goal (Ramnani and Owen, 2004).

### Deception through telling the truth (sophisticated deception)

Notably, finding this activation pattern both for simple as well as sophisticated deception trials, reveals that sophisticated deception, although superficially appearing as truth trials, cannot be considered a variant of plainly telling the truth—in which case no activation differences between sophisticated deception and truth trials should have occurred. Rather, the intention to deceive seems to share a lot with deceptive behavior in terms of cognitive processes. Sophisticated deception, as defined in the context of our sender-receiver game, is a form of deception that crucially has to take into account the receiver's reasoning. The sender has to form expectations about the receiver's beliefs and has to adjust her own actions accordingly. Hence, rTPJ activation becomes characteristic for sophisticated deception. Based on this finding, we suggest that brain activation can reveal the sender's veridical intention to deceive in the absence of overt lying. Accordingly, it seems warranted not to confine deception simply to telling a lie.

Interestingly, sophisticated deception seems also to stand out from simple deception. That is, trying to deceive the interaction partner by telling the truth requires greater processing demands than simply telling a lie. Particularly, given activation within the TPJ, lSTG, and MTG, we take this result to indicate greater demands when reading or inferring the partner's thoughts and beliefs so as to correctly predict the receiver's actions. That is, sophisticated deception differs from plainly telling a lie by heightened demands for ToM processes. Instead of construing additional activation (for instance within the frontal gyrus), our result may be understood as representing increasingly more complex processing of the social situation in strategic interaction (Bahnemann et al., 2010).

A further indication that simple and sophisticated deception are two different forms of deceptive behavior come from the parametric analysis. Only for simple deception trials part of the respective network was modulated by the distribution of monetary payoffs between sender and receiver. That is, activation within the dorsal ACC, amPFC, and aFG correlated positively the higher the conflict between sender's and receiver's payoffs. Activation within the dACC has consistently been related to conflict detection and monitoring processes (Carter and van Veen, 2007), although “conflict monitoring may be just one facet of the broader role of ACC in performance monitoring and the optimization of behavior” (Yeung, 2014, p. 275). Carter and van Veen (2007) suggested the ACC's specific role is “to detect conflict between simultaneous active, competing representations and to engage the dorsolateral prefrontal cortex (DLPFC) to resolve such conflict” (p. 367). The greater involvement of this area for high conflict trials when sending false messages may indicate greater tension in situations where people resort to lying despite knowing of the normative appeal to tell the truth.

### Genuine truth trials

By taking into account the sender's veridical intention, we could determine the neural correlates of genuine truth trials in the present study. Hitherto, imaging studies on deceptive behavior did not report any significant activation for telling the truth, which could be due to truth trials being a heterogeneous category in those studies, potentially also including sophisticated deception trials. We found significant activation within the habenular complex bilaterally and the left frontal operculum and MTG. Based on animal research, the habenular complex has been suggested to be specifically involved in the control of the human reward system. For instance, the electrical stimulation of the habenular nuclei resulted in an inhibition of up to 90% of the dopamine neurons in the ventral tegmental area and substantia nigra in rats (Christoph et al., 1986). In contrast, lesions to the habenular complex resulted in an “increased dopamine turnover in the nucleus accumbens, striatum, and prefrontal cortex, reflecting an activation of the dopaminergic system (Lisoprawski et al., 1980; Nishikawa et al., 1986)” (Ullsperger and von Cramon, 2003, p. 4308). Based on these as well as anatomical data, it has been suggested that the habenular complex serves as a “critical modulatory relay between the limbic forebrain structures and the midbrain” (Ullsperger and von Cramon, 2003, p. 4309). Accordingly, habenular activation for telling the truth in strategic interactions in the present study may reduce the probability of phasic dopamine release in the reward system, and thus may reinforce truth telling through weakening the incentive of the monetary profits.

In sum, our study provides a new paradigm for studying the neural basis of deception in human interaction. Contrary to previous studies with instructed deception in non-interactive contexts, we have created a social interactive context based on game-theoretic modeling. Importantly, we are the first to investigate the neural foundations of an intention to deceive in the absence of overt lying. Such sophisticated deception through telling the truth is an intriguing alternative to telling a plain lie, and it can be strategically used, as in the Austrian writer Franz Grillparzer's comedy “Woe to him who is lying” in which the young kitchen boy Leon frees his bishop's captured nephew by telling the guards he is going to free their hostage, and they let him proceed because they don't believe him.

### Conflict of interest statement

The authors declare that the research was conducted in the absence of any commercial or financial relationships that could be construed as a potential conflict of interest.

## Footnotes

^1^In recent years, a vast number of imaging studies have shown that there are marked differences in the neural localization of cognitive (and especially language) functions in the brains of left-handed individuals when compared with right-handers. To exclude a putative effect of lateralization correlated with handedness we had chosen to only include right-handers in our imaging study.
