# Supplementary material for: The neural basis of deception in strategic interactions
Source: Front Behav Neurosci. 2015 Feb 12;9:27. doi: 10.3389/fnbeh.2015.00027 (PMC4325923; doi:10.3389/fnbeh.2015.00027)
Supplement: Supplementary file 1 [file DataSheet1.DOCX]

**Appendix**

**A.1 Instructions for the sender (being scanned)**

Dear Participant!

Today, you are going to participate in the following experiment:

On the screen you will be presented with a table that might look like this one:

5 1

1 5

Myself

Player-2

Player-2 cannot see the numbers in this table, which correspond to Euros. Player-2 will have to choose either the red or blue column for implementation.

Before Player-2 does so, you can send a message to Player-2 that states which of the columns (blue or red) is more profitable for Player-2. Note that Player-2 does not get any other information except your message.

If Player-2 decides to choose the blue column, you will receive the amount of Euros that are indicated in the upper blue box (i.e., 1 Euro in this example) and Player-2 will get the amount of Euros that are indicated in the lower blue box (i.e., 5 Euros in this example).

If Player-2 decides to choose the red column, you will receive the amount of Euros that are indicated in the upper red box (i.e., 5 Euros in this example) and Player-2 will get the amount of Euros that are indicated in the lower red box (i.e., 1 Euro in this example).

Thus, you will always get one of the amounts that are displayed in the upper half of the table and Player-2 will always get one of the amounts displayed in the lower half of the table. The choice of Player-2 will determine whether the red or the blue column is relevant for payment.

**An Example:** You are sending a message to Player-2 that the blue column is more profitable for Player-2. Consider Player-2 indeed picks the blue column. As a result, you would receive 1 Euro and Player-2 would receive 5 Euros.

**Procedure**

You have two buttons to indicate which message you would like to send:

If you would like to send the message “The blue column is more profitable for you”, then press the blue button.

If you would like to send the message “The red column is more profitable for you”, then press the red button.

Please note again, Player-2 does not learn about the specific amounts, but can only choose between the blue and the red column.

After each decision, Player-2 gets the information how many Euros he or she has received in the specific trial. Yet, Player-2 does not learn how much she would have received had she opted for the other column. Neither does she learn about how many Euros you actually had received or how many points you would have received in the other column. Therefore, Player-2 will never be able to judge whether you sent a correct or an incorrect message.

Please note: it is important that you do not ponder for too long for each decisions, but decide within eight seconds, since the response buttons become inactive after eight seconds and the game automatically proceeds. If you accidentally miss one trial, this is not dramatic, but you should try to respond within the eight seconds.

After your choice for sending one of the two messages (and after eight seconds), you will be asked “What do you think which column (blue or red) is Player-2 going to choose?” You can indicate your response again by pressing the left (blue) or right (red) response button. You have four seconds for this response before the experiment automatically proceeds.

All your responses are recorded and after you finished the experiment they will be presented to Player-2 outside the scanner. Being informed about your message for each trial, Player-2 will have to decide on each trial whether to go for blue or red. Player-2 therefore never learns about the allocation of Euros in a particular trial; she only received your message. Thus, Player-2 cannot judge whether your message is correct or incorrect.

By the time when Player-2 finished the experiment, one trial will randomly be selected and paid to both of you accordingly. So, you could get as much as 30 Euros maximum!

If you have questions, please do not hesitate to ask them now, otherwise we start with the training session.

**A.2 Instructions for the receiver (recipient of the sender’s messages)**

Dear Participant!

Another player (Player-1) has just finished part 1 of our experiment. In this part, Player-1 was presented with various tables, which differed in the amount of Euros (indicated as numbers in the table) that could be obtained on each trial. Here is an example:

Player-2

Myself

5 1

1 5

Player 2

The upper half of the table corresponds to the profits Player-1 could get and the lower half to the profits you could get. Hence, you are in the role of Player-2 in this experiment. The profits of both players are linked such that always only ONE column (blue OR red) can be valid while the other becomes obsolete. Which of the two columns is valid entirely depends on YOUR choice, albeit you will never be presented with the specific tables and their associated potential profits.

In contrast, Player-1 is presented with the specific tables and the associated potential profits; yet, Player-1 is not authorized to decide which of the columns will become valid and hence which profits the two of you may receive. However, Player-1 can send you a message whether the blue or the red column is more profitable for you. The messages by Player-1 in each single trial were recorded and stored and will now be loaded accordingly for your decision trials (which we call rounds on the screen).

Here is an example screen:

Round: 1

Player-1 sent the following message:

The blue column is more profitable for you.

Which column do you choose?

(Geht das auch nicht eingrückt bzw. etwas schöner vom layout?)

If you press the blue button, the choice for the blue column is logged in and vice versa for the red button. Once you pressed a button, the next round starts, in which you are asked to make a new decision on which column to choose. Overall, there are 90 trials.

Once more, over the course of the experiment, you will neither learn about the various profits associated with the 90 tables nor about the specific distribution of the profits for you (Player-2) and Player-1 (e.g., identical or opposed profits). Just note, that the maximum profits for you or Player-1 are 30 Euros.

If you decide upon the blue (red) column, the profits in the blue (red) column are paid out to you and Player-1. But note that not all trials are paid out! Once you finished the 90 decisions, only ONE trial is randomly selected and paid to both of you accordingly.

Thank you very much for your attention and enjoy!

**A.3 Overview of the full set of games**

Listed are all matrices that were employed in the sender-receiver paradigm classified by category. Sender Red: payoff for the sender when state Red is chosen, sender Blue: payoff for the sender when state Blue is chosen, receiver Red: payoff for the receiver when choosing state Red, receiver Blue: payoff for the receiver when choosing state Blue (see also Figure 1 in the manuscript).

**Table A1**

Listed are all matrices in category “conflict” (n=45).

| Sender Red | Receiver Red | Sender Blue | Receiver Blue |
| --- | --- | --- | --- |
| 1 | 0 | 0 | 1 |
| 5 | 6 | 6 | 5 |
| 11 | 10 | 10 | 11 |
| 16 | 15 | 15 | 16 |
| 20 | 21 | 21 | 20 |
| 5 | 0 | 0 | 5 |
| 5 | 10 | 10 | 5 |
| 15 | 10 | 10 | 15 |
| 20 | 15 | 15 | 20 |
| 20 | 25 | 25 | 20 |
| 0 | 10 | 10 | 0 |
| 15 | 5 | 5 | 15 |
| 10 | 20 | 20 | 10 |
| 25 | 15 | 15 | 25 |
| 20 | 30 | 30 | 20 |
| 0 | 5 | 1 | 0 |
| 5 | 10 | 6 | 5 |
| 10 | 15 | 11 | 10 |
| 16 | 15 | 15 | 20 |
| 20 | 25 | 21 | 20 |
| 1 | 0 | 0 | 10 |
| 5 | 15 | 6 | 5 |
| 10 | 20 | 11 | 10 |
| 15 | 25 | 16 | 15 |
| 21 | 20 | 20 | 30 |
| 5 | 0 | 0 | 10 |
| 10 | 5 | 5 | 15 |
| 15 | 10 | 10 | 20 |
| 20 | 15 | 15 | 25 |
| 20 | 30 | 25 | 20 |
| 0 | 1 | 5 | 0 |
| 5 | 6 | 10 | 5 |
| 15 | 10 | 10 | 11 |
| 20 | 15 | 15 | 16 |
| 20 | 21 | 25 | 20 |
| 0 | 1 | 10 | 0 |
| 15 | 5 | 5 | 6 |
| 10 | 11 | 20 | 10 |
| 15 | 16 | 25 | 15 |
| 30 | 20 | 20 | 21 |
| 10 | 0 | 0 | 5 |
| 15 | 5 | 5 | 10 |
| 10 | 15 | 20 | 10 |
| 15 | 20 | 25 | 15 |
| 30 | 20 | 20 | 25 |

**Table A2**

Listed are all matrices in category “sender indifferent” (n=27).

| Sender Red | Receiver Red | Sender Blue | Receiver Blue |
| --- | --- | --- | --- |
| 1 | 0 | 1 | 1 |
| 6 | 5 | 6 | 6 |
| 11 | 10 | 11 | 11 |
| 1 | 5 | 1 | 0 |
| 6 | 10 | 6 | 5 |
| 11 | 15 | 11 | 10 |
| 1 | 0 | 1 | 10 |
| 6 | 15 | 6 | 5 |
| 11 | 20 | 11 | 10 |
| 5 | 0 | 5 | 5 |
| 10 | 5 | 10 | 10 |
| 15 | 10 | 15 | 15 |
| 5 | 10 | 5 | 0 |
| 10 | 15 | 10 | 5 |
| 15 | 20 | 15 | 10 |
| 5 | 0 | 5 | 15 |
| 10 | 5 | 10 | 20 |
| 15 | 10 | 15 | 25 |
| 10 | 10 | 10 | 0 |
| 15 | 15 | 15 | 5 |
| 20 | 20 | 20 | 10 |
| 10 | 0 | 10 | 15 |
| 15 | 5 | 15 | 20 |
| 20 | 25 | 20 | 10 |
| 10 | 0 | 10 | 20 |
| 15 | 5 | 15 | 25 |
| 20 | 30 | 20 | 10 |

**Table A3**

Listed are all matrices in category “aligned interest” (n=18).

| Sender Red | Receiver Red | Sender Blue | Receiver Blue |
| --- | --- | --- | --- |
| 1 | 1 | 0 | 0 |
| 0 | 0 | 1 | 5 |
| 1 | 10 | 0 | 0 |
| 5 | 5 | 6 | 6 |
| 6 | 10 | 5 | 5 |
| 5 | 5 | 6 | 15 |
| 10 | 10 | 11 | 11 |
| 11 | 15 | 10 | 10 |
| 10 | 10 | 11 | 20 |
| 5 | 1 | 0 | 0 |
| 0 | 0 | 5 | 5 |
| 5 | 10 | 0 | 0 |
| 5 | 5 | 10 | 6 |
| 10 | 10 | 5 | 5 |
| 5 | 5 | 10 | 15 |
| 10 | 10 | 15 | 11 |
| 15 | 15 | 10 | 10 |
| 15 | 20 | 10 | 10 |
